# Metabolomics analysis of children with autism, idiopathic-developmental delays, and Down syndrome

**DOI:** 10.1038/s41398-019-0578-3

**Published:** 2019-10-03

**Authors:** Jennie Sotelo Orozco, Irva Hertz-Picciotto, Leonard Abbeduto, Carolyn M. Slupsky

**Affiliations:** 1 Department of Nutrition, University of California, Davis, CA 95616 USA; 2 Department of Public Health Sciences, University of California, Davis, CA 95616 USA; 3 Department of Psychiatry and Behavioral Sciences, University of California, Davis, CA 95616 USA; 4 MIND Institute, University of California, Davis, CA 95817 USA; 5 Department of Food Science and Technology, University of California, Davis, CA 95616 USA

**Keywords:** Physiology, Autism spectrum disorders

## Abstract

Although developmental delays affect learning, language, and behavior, some evidence suggests the presence of disturbances in metabolism are associated with psychiatric disorders. Here, the plasma metabolic phenotype of children with autism spectrum disorder (ASD, *n* = 167), idiopathic-developmental delay (i-DD, *n* = 51), and Down syndrome (DS, *n* = 31), as compared to typically developed (TD, *n* = 193) controls was investigated in a subset of children from the case–control Childhood Autism Risk from Genetics and the Environment (CHARGE) Study. Metabolome profiles were obtained using nuclear magnetic resonance spectroscopy and analyzed in an untargeted manner. Forty-nine metabolites were identified and quantified in each sample that included amino acids, organic acids, sugars, and other compounds. Multiple linear regression analysis revealed significant associations between 11 plasma metabolites and neurodevelopmental outcome. Despite the varied origins of these developmental disabilities, we observed similar perturbation in one-carbon metabolism pathways among DS and ASD cases. Similarities were also observed in the DS and i-DD cases in the energy-related tricarboxylic acid cycle. Other metabolites and pathways were uniquely associated with DS or ASD. By comparing metabolic signatures between these conditions, the current study expands on extant literature demonstrating metabolic alterations associated with developmental disabilities and provides a better understanding of overlapping vs specific biological perturbations associated with these disorders.

## Introduction

It is estimated that about one in six children between the ages of 3 and 17 years in the United States have one or more developmental disability^1^. Developmental delays may impact day-to-day functioning, and usually last throughout a person’s lifetime. Some developmental delays have a known cause, such as the atypical cell division resulting in an extra portion of chromosome 21 (HSA21) that results in Down syndrome (DS). However, most developmental disabilities are thought to be caused by a combination of factors that include genetics and/or environment. One such example is autism spectrum disorder (ASD), which has been linked with genetic mutations and environmental exposures and thus has a complex gene-environmental origin^2,3^.

Although developmental delays are a group of conditions specifically affecting learning, language, and behavior, there is evidence that disturbances in metabolism may also be present^4^. ASD has been associated with increased oxidative stress^5,6^, decreased methylation capacity^6,7^, impaired sulfur metabolism^8,9^, gut microbiome dysbiosis^10,11^, and altered energy metabolism^12,13^. Comorbidities of trisomy HSA21 include overexpression of amyloid protein that results in increased Alzheimer’s risk^14^ as well as overexpression of superoxide dismutase 1 that results in the increased oxidative stress observed in individuals with DS^15^. Although, gene products of HSA21 may also be interacting with genes/proteins on other chromosomes resulting in widespread metabolic consequences. Interestingly, a set of dizygotic twins (one with DS, and the other with autism) was reported to have similar alterations in the methionine cycle and transsulfuration pathways^6^.

Metabolomics, the profiling of small-molecule metabolites, provides a tool to define perturbations in metabolic pathways. Indeed, the metabolome reflects the interaction between genetic and environmental influences and therefore can provide information to bridge the gap between genotype and phenotype. In this study, the plasma metabolome profiles of children (*n* = 442) diagnosed with ASD, DS, or idiopathic-developmental delay (i-DD), were compared to age-matched typically developed (TD) children. The objective of this study was to determine if there are identifiable plasma metabolome differences and commonalities associated with developmental delays of different etiologies.

## Subjects and methods

### Study population

All children in the present study are a subset of the Childhood Autism Risk from Genetics and Environment (CHARGE) Study^16^. The CHARGE study is a population-based case–control study which aims to uncover the environmental causes of autism and examine genetic factors and the interactions between genes and environment in the etiology of autism. Details about the study have previously been published^16^. Eligible children met the following criteria: (a) aged between 24 and 60 months at recruitment, (b) living with a biological parent who speaks English or Spanish, (c) born in California, and (d) residing in the study catchment areas. Participants were sampled from three strata: children with ASD, children with a developmental delay but not ASD (DD), and children from the general population (TD). ASD and DD children were recruited from the State of California Department of Developmental Services (DDS). The primary aim of CHARGE was to investigate ASD, and therefore TDs were matched for frequencies on age, sex, and broad geographic distribution of the autism cases. Each child’s diagnosis (ASD, DD, or TD) was confirmed at the UC Davis MIND (Medical Investigation for Neurodevelopmental Disorders) Institute (Sacramento, CA) as previously reported^16^.

**Fig. 1:**
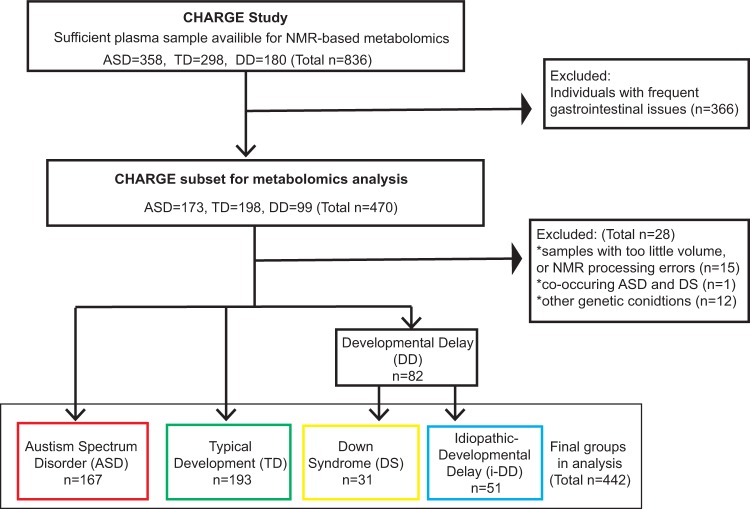
Flow chart of the study population

The sample size used in this study was based on a prior power test with some knowledge from previous literature. Using Bonferroni correction to account for multiple comparison (taking into account ~340 metabolites available in the Chenomx Profiler library, at *α* = 0.05, yielded an adjusted *α* of 0.0001), a total sample size of 300 (with equal numbers of each child’s diagnosis) would provide >99.9% power to detect a 1 SD difference; 94.7% power to detect a 0.8 SD difference, and 89% for a 0.75 SD difference for each pairwise comparison (e.g., ASD vs TD). Other choices for distributing the 300 samples (e.g., 130 ASD, 80 DD, 90 TD) would also yield adequate power even in the DD vs TD comparison, e.g., >80% power down to a 0.8 SD difference. A total of 836 children from CHARGE were considered for this analysis who had sufficient ACD plasma for metabolomics analysis. Individuals with frequent gastrointestinal symptoms were excluded (*n* = 366) from further analysis. Additionally, children with other genetic conditions (*n* = 12), too little volume or NMR processing errors (*n* = 15), and co-occurring conditions (*n* = 1) were also excluded. Furthermore, the original DD group in CHARGE was subdivided into an idiopathic DD (i-DD) group (with an unknown etiology) and a DS group (which was reported by a parent). Our final sample size was increased from the original power calculations to a total of 442 children (167 ASD, 51 i-DD, 31 DS, and 193 TD) due to the increased availability of samples and reduced cost of analysis (Fig. 1).

The CHARGE study was approved by the State of California Department of Developmental Services and the institutional review boards at the University of California, Davis, and Los Angeles. Informed consent was obtained prior to participation and any collection of data.

### ^1^H nuclear magnetic resonance (NMR) metabolomics analysis

Whole blood from each child was collected in yellow top (Acid Citrate Dextrose) tubes (BD Biosciences, San Jose, CA) at the time of child’s enrollment. Plasma was isolated and frozen at −80 °C until further analysis. For metabolomics analysis, thawed samples were filtered through an Amicon 3000 MW cut-off Centrifugal Device to remove lipids and proteins. The water-soluble filtrate was collected, and volume was adjusted with Type I ultrapure water from Millipore Synergy UV system (Millipore, Billerica, MI) if insufficient sample was collected. An internal standard (ISTD) containing DSS-D6 ([3-(trimethylsilyl)-1-propanesulfonic acid-d6], 0.2% NaN_3_, in 99.8% D_2_O) was added, and the pH of each sample was adjusted to 6.8 ± 0.1 by adding small amounts of NaOH or HCl. Volumes of HCl and NaOH added were recorded. An aliquot of the mixture was transferred to a labeled 3 mm Bruker NMR tube and stored at 4 °C until NMR acquisition (within 24 h of sample preparation).

Samples were run on a Bruker AVANCE 600 MHz NMR spectrometer equipped with a SampleJet autosampler using the NOESY-presaturation pulse sequence (noesypr). NMR spectra were acquired at 25 °C, with water saturation of 2.5 s during the prescan delay, a mixing time of 100 ms, 12 ppm sweep width, an acquisition time of 2.5 s, 8 dummy scans, and 32 transients. All spectra were zero-filled to 128 K data points and Fourier transformed with a 0.5 Hz line broadening applied. Spectra were manually phased and baseline-corrected and metabolites were identified and quantified using NMR Suite v8.1 (Chenomx Inc., Edmonton, Canada)^17^. The Chenomx profiler is linked to a database containing metabolite NMR spectral signatures encoded at different spectrometer ^1^H frequencies, including the 600 MHz containing 339 metabolites in its library valid at a pH between 4 and 9 (ideally close to 7). To ensure metabolite cluster fits remained within valid Chenomx profiler pH, the average pH was collected ((adjusted pH−pH post-NMR)/2) and manually input for each sample during sample processing. The average pH in all of our samples was 7.06 ± 0.3, ensuring all spectra were within a valid pH range for the quantification of metabolites. Comparison of the spectral data obtained for each plasma sample with the Chenomx metabolite library resulted in a list of compounds together with their respective concentrations based on the known concentration of the added internal standard. All compounds in the database have been verified against known concentrations of reference NMR spectra of the pure compounds and have been shown to be reproducible and accurate^18–20^. Neurodevelopmental diagnosis remained blinded from investigators during sample preparation as well as NMR data acquisition and analysis.

### Statistical analysis

Demographic characteristics of TD controls were compared to each of the ASD, i-DD, and DS case groups using the chi-square test for categorical variables and ANOVA for continuous variables. Metabolite concentrations (µM) were adjusted for any dilutions and log transformed because of the wide variation and skewed distribution. A total of 59 metabolites of diverse chemical classes were identified in plasma samples. However, metabolites identified in samples but originating from sample preparation (e.g. glucose, citrate, ethanol, glycerol), or falling below the detection limit for at least 20% of samples (e.g. fructose, maltose, beta-alanine, propionate, mannose, isopropanol) were excluded in the final analysis. Therefore a total of 49 metabolites were analyzed in this study and included those involved in amino acid metabolism, glutathione metabolism, glycolysis, homocysteine metabolism, ketone body synthesis, lipid metabolism, tricarboxylic acid (TCA) cycle, urea cycle, and others.

Multiple linear regression (MLR) (g*lm* function) was performed for each metabolite to assess the association between neurodevelopmental diagnosis (independent variables) and plasma metabolites (dependent variable). TD children were used as a reference group for the neurodevelopmental outcome. Possible confounders for neurodevelopmental diagnosis and metabolite concentrations were explored through a literature review and directed acyclic graphs (DAG) prior to model-building (Supplementary Fig. 1). Covariates considered in our DAG were child’s sex, child’s race/ethnicity, child’s age, child’s year of birth, maternal age at child’s birth, maternal race/ethnicity, maternal birthplace, year of blood collection, most recent food intake, and attributes pertaining to maternal socioeconomic variables such as parental homeownership and maximum maternal education. From the DAG, we then identified a sufficient set of adjustment factors that would remove confounding and minimize bias in the estimated associations between diagnostic group and metabolites. These included child’s sex (categorical: male/female [reference]), child’s race/ethnicity (categorical: Hispanic, Other, White [reference]), child’s age (continuous), year of plasma collection (categorical: 2003–2005, 2006–2008 [reference], 2009–2014), and parental homeownership (categorical: yes [reference], no). To account for multiple testing, we adjusted *p* values from regression analysis by controlling the false discovery rate (FDR) at 5% using the Benjamini–Hochberg procedure (*p.adjust.method* = *"BH"*) with *p*_FDR_ values < 0.05 as statistically significant. Effect size between ASD vs TD, i-DD vs TD, and DS vs TD was evaluated using Cliff’s delta (*δ*) statistic (*cliff.delta* function from the *effsize* package). |*δ*| < 0.33 corresponds to small, |*δ*| < 0.474 corresponds to medium, and |*δ*| > 0.475 corresponds to large effect size in metabolite concentration differences. KEGG (Kyoto Encyclopedia of Genes and Genomes) pathway database (http://www.genome.ad.jp/kegg/), HMDB (Human metabolome database)^21^ (www.hmdb.ca), and MetaboAnalyst Web application (www.metaboanalyst.ca) were used to further examine the identified metabolic pathways. Statistical analyses were performed using R 3.3.3 (R Foundation for statistical computing, version 3.0.1, Vienna Austria. URL http://www.R-project.org) and SAS software version 9.4 (SAS Institute Inc).

## Results

General characteristics of participants in this study are presented in Table 1. Children with ASD tended to be socio-demographically similar to TD children. I-DD and particularly DS children were more likely to be female than were TD children. This was because the TD sex distribution was matched to the projected ASD sex ratio of 4:1 (males:females), but the DD group was not matched. I-DD cases were more likely to be Hispanic and their mothers tended to be the least educated. A larger proportion of mothers of children with DS and TD were homeowners compared to mothers of children with ASD and i-DD. The majority of mothers were born in the US. Additionally, as expected, mothers of children with DS tended to be older.

**Table 1:** Sociodemographic of the study population

Characteristics of study population	ASD cases (*N* = 167)	i-DD cases (*N* = 51)	DS cases (*N*=31)	TD controls (*N* = 193)	*P* value^a^
Child’s sex, % (n)					
Male	73.65 (123)	58.82 (30)	35.48 (11)	73.06 (141)	<0.0001^a^
Female	26.35 (44)	41.18 (21)	64.52 (20)	26.94 (52)	
Child’s age (months)					
Mean (SD)^b^	43.96 (10.23)	46.67 (7.88)	44.41 (9.18)	43.09 (9.76)	0.13^a^
Child’s race/ethnicity, % (n)					
White	44.91 (75)	39.22 (20)	48.39 (15)	52.33 (101)	0.22^a^
Hispanic	35.53 (61)	43.41 (22)	38.71 (12)	26.42 (41)	
Other	18.56 (31)	17.65 (9)	12.90 (4)	21.24 (41)	
Year of blood collection, % (n)					
2003–2005	30.54 (51)	7.84 (4)	3.23 (1)	13.47 (26)	0.0002^a^
2006–2008	32.34 (54)	37.25 (19)	35.48 (11)	37.82 (73)	
2009–2011	21.56 (36)	39.22 (20)	32.26 (10)	31.61 (61)	
2012–2014	15.57 (26)	15.69 (8)	29.03 (9)	17.10 (33)	
Maximum maternal education in home, % (n)					
High school graduate or less	18.56 (31)	17.65 (9)	16.13 (5)	14.51 (28)	0.37^a^
Some college or technical, vocational or associate degree	35.33 (59)	50.98 (26)	32.26 (10)	32.64 (63)	
Bachelor’s degree	34.13 (57)	25.49 (13)	41.94 (13)	38.86 (75)	
Graduate or professional degree	11.98 (20)	5.88 (3)	9.68 (3)	13.99 (27)	
Maternal birthplace					
US	74.85 (125)	78.43 (40)	80.25 (25)	84.97 (164)	0.24^a^
Mexico	9.58 (16)	7.84 (4)	9.68 (3)	3.63 (7)	
Other	15.57 (26)	13.73 (7)	9.68 (3)	11.40 (22)	
Maternal age at child’s birth (years), % (n)					
≤19	3.59 (6)	3.92 (2)	0.00 (0)	5.18 (10)	0.005^a^
20–29	37.12 (62)	49.02 (25)	29.03 (9)	36.79 (71)	
30–34	31.14 (52)	23.53 (12)	12.90 (4)	36.27 (70)	
≥35	28.14 (47)	23.53 (12)	58.06 (18)	21.76 (42)	
Homeownership, % (n)					
Yes	63.47 (106)	60.78 (31)	70.97 (22)	74.09 (143)	0.10^a^
No	36.53 (61)	39.22 (20)	29.03 (9)	25.91 (50)	

^a^*X*^2^ test (nominal data) or ANOVA test (continuous data) was performed

^b^*SD* standard deviation

MLR analysis (Table 2) demonstrated significant associations between 28 plasma metabolites and neurodevelopment when comparing ASD, i-DD, and DS cases to TD controls adjusting for child’s sex, child’s race/ethnicity, child’s age, year of blood collection, and parental homeownership. Eleven metabolites remained significant after FDR correction.

**Table 2:** Multiple linear regression (MLR) analysis of log-transformed plasma metabolites concentrations for children with autism spectrum disorder (ASD; *n* = 167), idiopathic-developmental delay (i-DD; *n* = 51), and Down syndrome (DS; *n* = 31), each compared to children with typical development (TD; *n* = 193)

Pathway	Metabolite	Diagnosis	*β*	95% CI	P _FDR_	*δ*
Bacterial metabolite	Acetate	ASD	−0.025	(−0.082, 0.033)	0.683	−0.01
		i-DD	0.109	(0.024, 0.193)	0.097	0.28
		DS	0.134	(0.029, 0.239)	0.097	0.37
	**Dimethyl sulfone**	ASD	−0.026	(−0.072, 0.020)	0.575	−0.17
		i-DD	0.012	(−0.056, 0.079)	0.903	0.05
		**DS**	**0.133**	(**0.049, 0.217)**	**0.030**	**0.87**
BCAA metabolism, amino acid metabolism	2-Hydroxyisovalerate	ASD	−0.023	(−0.055, 0.008)	0.407	−0.18
		i-DD	−0.040	(−0.086, 0.006)	0.288	−0.28
		DS	0.009	(−0.048, 0.066)	0.913	0.07
	3-Hydroxyisobutyrate	ASD	−0.057	(−0.101, −0.014)	0.094	−0.26
		i-DD	−0.020	(−0.084, 0.044)	0.839	−0.13
		DS	0.038	(−0.042, 0.117)	0.618	0.15
	3-Methyl-2-oxovalerate	ASD	0.003	(−0.030, 0.035)	0.956	−0.02
		i-DD	0.010	(−0.037, 0.057)	0.861	0.02
		DS	0.051	(−0.007, 0.110)	0.286	0.32
	Isoleucine	ASD	0.007	(−0.024, 0.039)	0.861	0.05
		i-DD	0.002	(−0.045, 0.049)	0.984	0.01
		DS	0.036	(−0.022, 0.094)	0.511	0.22
	Leucine	ASD	0.004	(−0.024, 0.032)	0.915	0.01
		i-DD	0.003	(−0.037, 0.044)	0.956	0.03
		DS	0.026	(−0.025, 0.076)	0.609	0.19
	Valine	ASD	−0.002	(−0.027, 0.024)	0.977	−0.04
		i-DD	−0.007	(−0.045, 0.031)	0.888	−0.08
		DS	0.041	(−0.006, 0.088)	0.286	0.32
Glutathione metabolism	2-Aminobutyrate	ASD	−0.034	(−0.062, −0.006)	0.113	−0.22
		i-DD	−0.014	(-0.056, 0.027)	0.795	−0.04
		DS	0.000	(−0.052, 0.051)	0.995	0.05
Glutathione metabolism, amino acid metabolism	2-Hydroxybutyrate	ASD	−0.054	(−0.098, −0.009)	0.113	−0.18
		i-DD	0.015	(−0.050, 0.080)	0.861	0.08
		DS	0.040	(−0.041, 0.121)	0.609	0.16
	Histidine	ASD	0.031	(0.010, 0.053)	0.051	0.16
		i-DD	−0.013	(−0.045, 0.018)	0.691	−0.12
		DS	−0.019	(−0.059, 0.020)	0.609	−0.16
Glutathione metabolism, lipid metabolism, glycine, serine, threonine metabolism, amino acid metabolism	**Serine**	**ASD**	**0.031**	(**0.010, 0.051)**	**0.039**	**0.25**
		i-DD	0.027	(−0.003, 0.057)	0.260	0.31
		DS	−0.006	(−0.043, 0.031)	0.913	0.04
Glycine, serine, threonine metabolism, amino acid metabolism	Threonine	ASD	0.020	(−0.008, 0.047)	0.426	0.12
		i-DD	0.020	(−0.021, 0.060)	0.609	0.18
		DS	0.027	(−0.024, 0.077)	0.589	0.24
Glycolysis	Lactate	ASD	0.053	(0.011, 0.095)	0.101	0.17
		i-DD	0.073	(0.011, 0.135)	0.122	0.41
		DS	0.071	(−0.006, 0.148)	0.260	0.48
	Pyruvate	ASD	−0.007	(−0.072, 0.058)	0.938	−0.08
		i-DD	−0.021	(−0.117, 0.074)	0.861	0.02
		DS	−0.042	(−0.160, 0.077)	0.787	0.00
Glycolysis, amino acid metabolism	**Alanine**	**ASD**	**0.040**	(**0.018, 0.062)**	**0.010**	**0.28**
		i-DD	0.019	(−0.013, 0.052)	0.540	0.20
		DS	0.024	(−0.017, 0.064)	0.546	0.35
Homocysteine metabolism	Betaine	ASD	0.029	(0.003, 0.056)	0.155	0.19
		i-DD	0.000	(−0.040, 0.039)	0.995	0.03
		DS	−0.016	(−0.064, 0.033)	0.839	−0.05
	***N*** **-** ***N*** **-Dimethylglycine**	ASD	0.017	(−0.018, 0.052)	0.618	0.06
		i-DD	−0.001	(−0.053, 0.051)	0.984	−0.03
		**DS**	**0.104**	(**0.039, 0.168)**	**0.028**	**0.67**
Homocysteine metabolism, glutathione metabolism, glycine, serine, threonine metabolism, amino acid metabolism	**Glycine**	**ASD**	**0.038**	(**0.015, 0.061)**	**0.023**	**0.24**
		i-DD	0.019	(−0.015, 0.052)	0.581	0.24
		DS	0.029	(−0.012, 0.071)	0.437	0.36
Homocysteine metabolism, lipid metabolism	**Choline**	ASD	0.027	(0.002, 0.051)	0.156	0.12
		i-DD	0.035	(−0.002, 0.071)	0.238	0.26
		**DS**	**0.104**	(**0.059, 0.149)**	**0.000**	**0.98**
Homocysteine metabolism, methionine cycle, amino acid metabolism	Methionine	ASD	0.019	(−0.020, 0.058	0.609	0.09
		i-DD	0.014	(−0.043, 0.071)	0.861	0.08
		DS	−0.011	(−0.082, 0.060)	0.913	−0.05
Ketone body	3-Hydroxybutyrate	ASD	−0.033	(−0.113, 0.046)	0.691	−0.02
		i-DD	0.026	(−0.091, 0.143)	0.861	0.01
		DS	0.133	(−0.011, 0.278)	0.260	0.23
	Acetoacetate	ASD	−0.027	(−0.130, 0.075)	0.861	−0.05
		i-DD	−0.198	(−0.349, −0.046)	0.094	−0.39
		DS	−0.136	(−0.324, 0.052)	0.414	−0.38
	Acetone	ASD	−0.028	(−0.062, 0.007)	0.342	−0.09
		i-DD	0.012	(−0.039, 0.063)	0.861	0.07
		DS	0.060	(−0.003, 0.123)	0.238	0.24
Lipid metabolism	**Carnitine**	ASD	−0.010	(−0.029, 0.010)	0.609	−0.17
		i-DD	0.018	(−0.011, 0.046)	0.511	0.25
		**DS**	**0.065**	(**0.030, 0.100)**	**0.009**	**0.82**
	***O*** **-Acetylcarnitine**	ASD	−0.008	(−0.046, 0.030)	0.861	0.01
		i-DD	0.029	(−0.026, 0.084)	0.589	0.19
		**DS**	**0.152**	(**0.083, 0.221)**	**0.000**	**0.82**
Lysine metabolism, amino acid metabolism	Lysine	ASD	0.006	(−0.021, 0.034)	0.861	0.01
		i-DD	0.002	(−0.039, 0.042)	0.984	0.08
		DS	0.059	(0.009, 0.109)	0.120	0.57
Neurotransmitter precursor amino acid, amino acid metabolism	Phenylalanine	ASD	0.005	(−0.016, 0.027)	0.861	−0.03
		i-DD	0.019	(−0.013, 0.051)	0.540	0.20
		DS	0.008	(−0.032, 0.047)	0.887	0.14
	Tyrosine	ASD	−0.004	(−0.035, 0.026)	0.916	−0.06
		i-DD	0.012	(−0.033, 0.057)	0.861	0.06
		DS	−0.047	(−0.103, 0.009)	0.306	−0.33
Neurotransmitter precursor amino acid, glutathione metabolism, amino acid metabolism	Glutamate	ASD	−0.015	(−0.069, 0.039)	0.861	−0.08
		i-DD	0.109	(0.030, 0.188)	0.076	0.42
		DS	0.059	(−0.040, 0.157)	0.540	0.28
Neurotransmitter precursor amino acid, glutathione metabolism, urea cycle, amino acid metabolism	Glutamine	ASD	0.026	(−0.009, 0.061)	0.407	0.09
		i-DD	−0.033	(−0.085, 0.019)	0.511	−0.13
		DS	−0.015	(−0.079, 0.049)	0.861	−0.03
Neurotransmitter precursor amino acid, tryptophan metabolism, amino acid metabolism	Tryptophan	ASD	0.038	(−0.019, 0.095)	0.487	0.21
		i-DD	0.008	(−0.076, 0.092)	0.947	0.06
		DS	0.005	(−0.099, 0.110)	0.977	0.00
One-carbon metabolism	Formate	ASD	−0.014	(−0.040, 0.012)	0.589	−0.01
		i-DD	0.018	(−0.020, 0.056)	0.627	0.09
		DS	−0.011	(−0.058, 0.036)	0.861	−0.24
Other, amino acid metabolism	Taurine	ASD	−0.004	(−0.029, 0.022)	0.914	−0.09
		i-DD	0.004	(−0.033, 0.041)	0.938	0.13
		DS	−0.026	(−0.072, 0.021)	0.581	−0.10
Others, proline metabolism	*Trans*-4-Hydroxy-l-proline	ASD	0.001	(−0.029, 0.031)	0.984	−0.03
		i-DD	0.000	(−0.044, 0.045)	0.995	0.06
		DS	0.021	(−0.034, 0.076)	0.746	0.21
Polyamines & creatine	Creatine	ASD	−0.001	(−0.025, 0.023)	0.977	−0.07
		i-DD	−0.019	(−0.054, 0.017)	0.589	−0.15
		DS	0.037	(−0.007, 0.081)	0.308	0.29
	**Creatinine**	ASD	−0.001	(−0.019, 0.017)	0.984	−0.02
		i-DD	−0.004	(−0.030, 0.023)	0.916	0.09
		**DS**	**0.078**	(**0.045, 0.111)**	**0.000**	**0.80**
Purine metabolism	Hypoxanthine	ASD	−0.053	(−0.146, 0.041)	0.581	−0.10
		i-DD	0.039	(−0.099, 0.177)	0.861	0.08
		DS	0.038	(−0.133, 0.209)	0.861	0.03
Sugar derivatives	Myo-inositol	ASD	0.007	(−0.023, 0.038)	0.861	−0.04
		i-DD	0.010	(−0.035, 0.054)	0.861	−0.01
		DS	0.075	(0.020, 0.131)	0.079	0.54
TCA cycle	**2-Oxoglutarate**	ASD	0.036	(−0.002, 0.073)	0.238	0.10
		i-DD	0.060	(0.005, 0.115)	0.155	0.37
		**DS**	**0.142**	(**0.074, 0.210)**	**0.000**	**0.93**
	*Cis*-aconitate	ASD	0.048	(0.007, 0.088)	0.123	0.17
		i-DD	0.065	(0.005, 0.126)	0.156	0.40
		DS	0.094	(0.019, 0.168)	0.101	0.53
	Fumarate	ASD	0.004	(−0.032, 0.040)	0.938	0.03
		i-DD	0.014	(−0.039, 0.066)	0.861	0.06
		DS	0.002	(−0.064, 0.067)	0.984	0.00
TCA cycle	Succinate	ASD	0.010	(−0.024, 0.044)	0.854	0.07
		i-DD	0.059	(0.009, 0.110)	0.120	0.41
		DS	0.049	(−0.013, 0.111)	0.352	0.45
Urea cycle	**Ornithine**	**ASD**	**0.051**	(**0.021, 0.081)**	**0.017**	**0.24**
		i-DD	0.033	(−0.011, 0.077)	0.392	0.28
		DS	0.035	(−0.020, 0.090)	0.511	0.37
	Urea	ASD	−0.028	(−0.058, 0.003)	0.276	−0.20
		i-DD	−0.036	(−0.081, 0.009)	0.352	−0.30
		DS	0.070	(0.014, 0.126)	0.101	0.43
Urea cycle, amino acid metabolism	Arginine	ASD	0.037	(0.001, 0.072)	0.184	0.23
		i-DD	0.020	(−0.033, 0.072)	0.757	0.13
		DS	0.034	(−0.031, 0.099)	0.589	0.25
	Asparagine	ASD	0.030	(0.001, 0.058)	0.173	0.11
		i-DD	−0.036	(−0.078, 0.006)	0.289	−0.24
		DS	0.007	(−0.045, 0.058)	0.926	0.12
	Proline	ASD	−0.002	(−0.032, 0.028)	0.965	−0.07
		i-DD	0.009	(−0.036, 0.053)	0.887	0.05
		DS	0.020	(−0.036, 0.075)	0.787	0.21
Xenobiotic	Glycolate	ASD	−0.025	(−0.050, 0.001)	0.238	−0.08
		i-DD	0.025	(−0.013, 0.063)	0.487	0.18
		DS	−0.010	(−0.057, 0.037)	0.861	−0.17

MLR models were adjusted for child’s sex, child’s race/ethnicity, child’s age at blood draw, parental homeownership, and year of blood collection. Beta-coefficients (*β*) and 95% confidence intervals (CI) are presented. Complete list of plasma metabolites identified and quantified in study is presented. The metabolites which remained significant after FDR correction (*P*_FDR_) at 5% are in bold. Effect size (*δ*) between ASD vs TD, i-DD vs TD, and DS vs TD is presented below: |*δ*| < 0.33 corresponds to small, |*δ*| < 0.474 corresponds to medium, |*δ*|>0.475 corresponds to large effect sizes

ASD cases had lower levels of 2-aminobutyrate (*β* = −0.034, *p* = 0.018), 2-hydroxybutyrate (*β* = −0.054, *p* = 0.018), and 3-hydroxyisobutyrate (*β* = −0.057, *p* = 0.010), but higher plasma concentrations of betaine (*β* = 0.029, *p* = 0.032), choline (*β* = 0.027, *p* = 0.034), *cis*-aconitate (*β* = 0.048, *p* = 0.023), lactate (*β* = 0.053, *p* = 0.014), ornithine (*β* = 0.051, *p* = 0.001), and the amino acids alanine (*β* = 0.040, *p* = 0.0001), arginine (*β* = 0.037, *p* = 0.043), asparagine (*β* = 0.030, *p* = 0.039), glycine (*β* = 0.038, *p* = 0.003), histidine (*β* = 0.031, *p* = 0.004), and serine (*β* = 0.031, *p* = 0.003) compared to TD controls. Only higher levels of alanine (*p*_FDR_ = 0.009), glycine (*p*_FDR_ = 0.022), ornithine (*p*_FDR_ = 0.016), and serine (*p*_FDR_ = 0.041) remained significant after FDR correction but had small effect sizes (|*δ*| < 0.33).

Children with i-DD had, similar to the ASD children, higher plasma *cis*-aconitate (*β* = 0.065, *p* = 0.033) and lactate (*β* = 0.073, *p* = 0.022); distinct from the ASD children, they also had higher plasma 2-oxoglutarate (*β* = 0.060, *p* = 0.032), acetate (*β* = 0.109, *p* = 0.012), glutamate (*β* = 0.109, *p* = 0.007), and succinate (*β* = 0.059, *p* = 0.021), but lower levels of acetoacetate (*β* = −0.198, *p* = 0.011) compared to TD controls. After FDR correction, none of the metabolites remained significant; however, trends for elevated plasma acetate, glutamate, lactate, and the TCA cycle intermediates (2-oxoglutarate, cis-aconitate, and succinate) were close to significant among i-DD cases as compared to controls.

Children with DS had higher plasma 2-oxoglutarate (*β* = 0.142, *p* < 0.0001), acetate (*β* = 0.134, *p* = 0.0102), carnitine (*β* = 0.065, *p* < 0.0001), *cis*-aconitate (*β* = 0.094, *p* = 0.014), choline (*β* = 0.104, *p* < 0.0001), creatinine (*β* = 0.078, *p* < 0.0001), dimethyl sulfone (*β* = 0.133, *p* = 0.002), lysine (*β* = 0.059, *p* = 0.020), myo-inositol (*β* = 0.075, *p* = 0.008), *N*,N-dimethylglycine (*β* = 0.104, *p* = 0.0002), *O*-acetylcarnitine (*β* = 0.152, *p* < 0.0001), and urea (*β* = 0.070, *p* = 0.015) compared to TD controls. After FDR adjustment, lipid metabolism metabolites carnitine (*p*_FDR_ = 0.008) and *O*-acetylcarnitine (*p*_FDR_ = 0.0004), homocysteine metabolism metabolites (*N*,*N*-dimethylglycine (*p*_FDR_ = 0.027) and choline (*p*_FDR_ = 0.0004)), TCA cycle intermediate 2-oxoglutarate (p_FDR_ = 0.0004), as well as creatinine (*p*_FDR_ = 0.0004), and dimethyl sulfone (*p*_FDR_ = 0.029) remained elevated in DS cases as compared to TD controls. Large effect sizes (|*δ*| > 0.475) were observed for these metabolites.

## Discussion

**Fig. 2:**
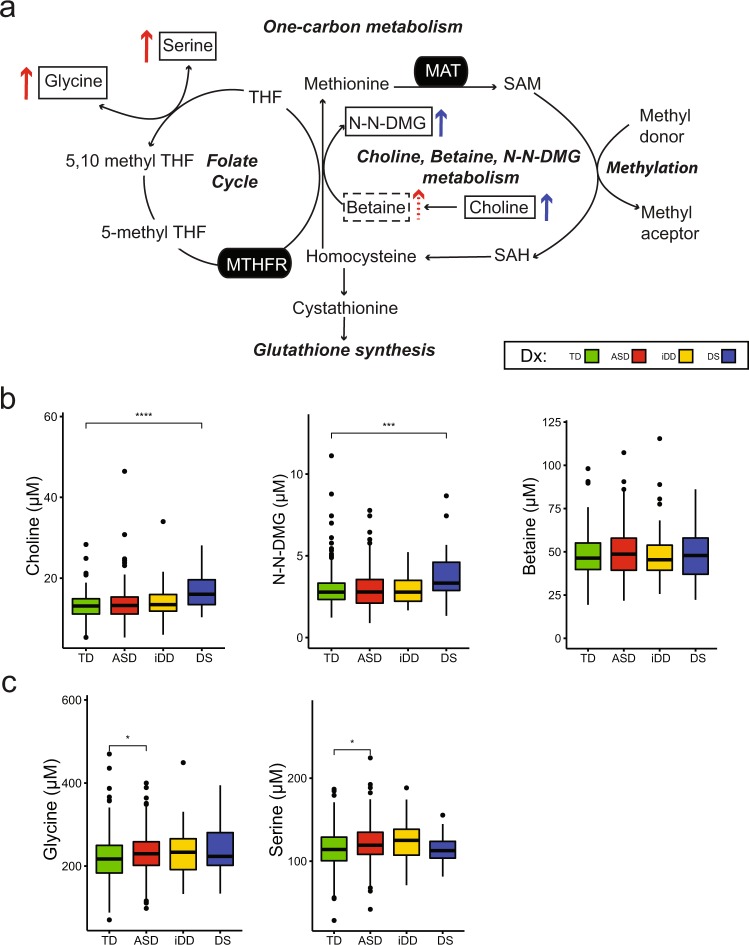
**a** One-carbon metabolism: metabolic pathways converging at homocysteine metabolism, glutathione biosynthesis, folate cycle, and choline/betaine metabolism. Colored arrows indicate differences in metabolite concentrations comparing several diagnostic groups to those with typical development. The color coding key is on the right side of the figure. Boxed metabolites were measured in this study. Solid boxes identify analytes significantly different after FDR correction, while dashed boxes identify those with a trend (i.e. significant unadjsuted *p* value) as compared to TD controls. Gray shading identifies metabolites that were not significantly different as compared to TD controls. Enzymes are represented in black ovals. **b** Boxplots of choline (μM), *N*,*N*-DMG (μM), and betaine (μM). **c** Boxplot of glycine (μM) and serine (μM). Side-by-side boxplots of metabolite concentrations (μM) display the distribution (range and interquartile range) across the different neurodevelopmental groups as compared to controls. The variance illustrated in the boxplots is similar across groups but within expectation given the sample size of each group for each metabolite. Significant *p* values (*< 0.05, **< 0.01, ***< 0.001) after controlling for Benjamin–Hochberg false discovery rate (*α* < 0.05) are presented. Dx diagnosis, MTHFR methylenetetrahydrofolate reductase, *N*-*N*-DMG: *N*-*N*-dimethylglycine, THF tetrahydrofolate, SAM *S*-adenosylmethionine, SAH *S*-adenosylhomocysteine

**Fig. 3:**
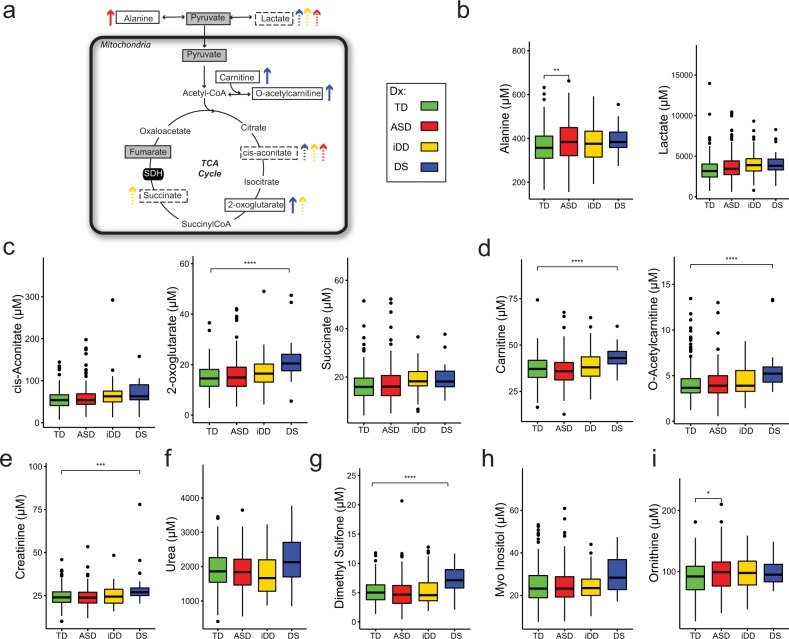
**a** Overview of the tricarboxylic acid (TCA) cycle. Colored arrows indicate differences in metabolite concentrations comparing several diagnostic groups to those with typical development. The color coding key is indicated on the figure. Boxed metabolites were measured in this study. Solid boxes identify analytes significantly different after FDR correction, while dashed boxes identify those with a trend (i.e. significant unadjsuted *p* value) as compared to TD controls. Gray shading identifies metabolites that were not significantly different as compared to TD controls. Enzymes are represented in black ovals. **b** Boxplots of alanine (μM) and lactate (μM). **c** Boxplots of cis-aconitate (μM), 2-oxoglutarate (μM), and succinate(μM). **d** Boxplots of carnitine (μM) and *O*-acetylcarnitine (μM). Boxplots of metabolite concentrations (μM) elevated in DS and ASD cases not already shown in one-carbon metabolism or the tricarboxylic acid (TCA) cycle: **e** creatinine (μM), **f** urea (μM), **g** dimethyl sulfone (μM), **h** myo-Inositol (μM), **i** ortnithine (μM). Side-by-side boxplots of metabolites concentrations (μM) display the distribution (range and interquartile range) across the different neurodevelopmental groups as compared to controls. The variance illustrated in the boxplots is similar across groups but within expectation given the sample size of each group for each metabolite. Significant *p* values (*< 0.05, **< 0.01, ***< 0.001) after controlling for Benjamin–Hochberg false discovery rate (*α* < 0.05) are presented. TCA tricarboxylic acid cycle, SDH succinate dehydrogenase

In this study, we investigated the plasma metabolomic profiles of children with ASD, i-DD, and DS compared to age-matched TD controls. Interestingly, we observed perturbation in one-carbon metabolism pathways among DS and ASD cases, although ASD was associated with the folic acid-folate cycle, whereas the methionine cycle was affected in DS (Fig. 2a). Similarly, DS, i-DD, and ASD cases each showed some differences from TD children in mitochondrial dysfunction and/or the TCA cycle, with the i-DD and DS tending to be the highest for several TCA cycle metabolites (Fig. 3a). However, other metabolites were uniquely associated with DS and ASD (Fig. 3e–i).

In comparison with TD controls, there were large effect size differences for children with DS, who had significantly elevated levels of choline and *N*,*N*-dimethylglycine (*N*,*N*-DMG) (Fig. 2b). A trend for elevated betaine was also found among ASD cases compared to controls. Choline and its oxidation product betaine can serve as methyl group donors during folate deficiency for one-carbon metabolism. Indeed, low folate availability has previously been shown to upregulate the choline dependent remethylation of homocysteine^22^, and a concomitant increase in blood *N*,*N*-DMG concentration^23^. Our results suggest upregulation of choline, betaine, and *N*,*N*-DMG pathway as methyl donors in DS and ASD is potentially related to folate availability. Obeid et al.^24^ reported similar findings in methylation pathway metabolites and found elevated blood cystathionine, cysteine, betaine, choline, and *N*,*N*-DMG in children and young adults with DS (*n* = 35) compared to age-matched controls (*n* = 47). Moreover, new one-carbon units primarily enter the folate cycle as 5,10-methylene-THF (5,10 methyl-THF) made from the choline/betaine/*N*,*N*-DMG metabolism pathway, or from the interconversion of the amino acids serine and glycine. Interestingly, we found elevated levels of glycine and elevated serine among ASD cases compared to controls (Fig. 2c). Glycine is the simplest amino acid with a number of functions including fat metabolism, neurological function, muscle development, and incorporation into the antioxidant glutathione^25^. Polymorphisms in the 5,10-methylenetetrahydrofolate reductase (MTHFR, EC 1.5.1.20) enzyme (MTHFR 677 C→T) affect folate availability^26^ and increase the need for other interdependent metabolites including choline and betaine^27^. Our results demonstating elevated glycine and serine provides additional evidence of the reduced ability of the folate cycle to utilize one-carbon units in ASD. Surprisingly, the MTHFR 677T alleles (with decreased enzymatic activity) have been found to be more prevalent among children with DS^28–30^ and among individuals with ASD^31–33^. There is also a higher frequency of MTHFR 677T alleles among Hispanics than in non-Hispanics^34,35^, which may be a contributing factor to the increased risk of DS reported among Hispanic populations^36^. Though a greater proportion of our DS cases were Hispanic compared to controls (Table 1), our final MLR models were adjusted for race/ethnicity. However, we did not account for MTHFR genotype, which may play a contributing factor.

Mitochondria are complex cellular organelles governing many metabolic processes including oxidative phosphorylation, the electron transport chain, fatty acid oxidation, the tricarboxylic (TCA) acid cycle, and many others. Elevated plasma alanine among ASD cases, in addition to trends for elevated lactate levels in children with DS, i-DD, and ASD, suggests peripheral mitochondrial dysfunction associated with these disorders (Fig. 3b). Indeed, these specific biochemical changes and mitochondrial dysfunction more generally have previously been reported in individuals with DS, motor delays, impaired neurological function, developmental delays, and ASD^15,37–42^. In addition, based on our observation of elevated levels of plasma 2-oxoglutarate, we identified abnormalities in the TCA cycle among DS cases (Fig. 3c). Morevover, there also was a trend for increased 2-oxoglutarate and succinate among i-DD cases, and a trend for elevated cis-aconitate among DS, i-DD, and ASD cases. The TCA cycle takes place in the mitochondrial matrix and is critical for converting carbohydrates and fats into cellular energy. Although the cause of developmental delays in i-DD cases is unknown, elevated levels of TCA cycle metabolites could play a role in symptomology and/or etiology. For example, inborn errors of metabolism (including inborn errors in the TCA cycle) often result in adverse neurodevelopment in affected individuals^43^. Collectively, these findings show that TCA cycle metabolism is altered in DS and i-DD cases, and potentially ASD cases which also had a trend for elevated cis-aconitate, and peripheral markers of mitochondrial dysfunction (lactate and alanine). Interestingly, we found a large effect size for elevated carnitine and *O*-acetylcarnitine (acetylated carnitine) in DS (Fig. 3d), suggesting altered mitochondrial fatty acid oxidation as these metabolites serve as shuttles in the mitochondria for fatty acid beta-oxidation. Overall, our findings provide evidence of similarly altered mitochondrial function in the TCA cycle in DS and i-DD cases. As well as altered carnitine metabolism among DS cases.

Other metabolites uniquely perturbed in DS included elevated creatinine and a trend for elevated urea compared to TD controls (Fig. 3e, f), possibly suggesting reduced kidney glomerular filtration. Indeed, elevated levels of blood creatinine and urea have previously been described in DS cases with reduced renal function^44^. Interestingly, nephropathy is a common complication of congenital heart disease^45^ (a condition which affects about 40–50% of individuals with DS^46^) and may play a contributing role. However, elevated creatinine may also be related to further evidence of altered methylation in DS, considering the production of creatine (creatinine’s precursor) from guanidinoacetate requires a methyl group from *S*-adenosyl-methionine (SAM). We observed DS cases also had significantly elevated levels of dimethyl sulfone compared to TD controls (Fig. 3g). Dimethyl sulfone can be derived from various sources including diet, human endogenous methanethiol metabolism, and intestinal microbiota metabolism^47^. Elevated dimethyl sulfone in plasma and cerebrospinal fluid was previously reported in patients with neurometabolic disease^48^, including patients with methionine adenosyltransferase I/III deficiency (MAT I/III)^48^ (the enzyme responsible for activating methionine to SAM in one-carbon metabolism^49^), again implicating reduced methylation capacity in DS. Of note, DS cases also had a trend for elevated levels of myo-inositol compared to controls (Fig. 3h). Myo-inositol can be obtained from the diet or be endogenously produced. It plays an important role as a second messenger^50^ and in the composition of phospholipids^51^. Elevated myo-inositol has previously been reported^52^ in elderly patients with Alzheimer’s disease (a common comorbidity in DS^53^), suggesting abnormalities in the inositol messenger pathway occur early in the etiology of Alzheimer’s. Collectively, the metabolites altered in DS cases appear to be related to methylation pathways, the TCA cycle, carnitine metabolism, or comorbidities common in DS.

We also found some evidence of altered amino acid metabolism among children with ASD. Elevated ornithine, a non-proteinogenic amino acid which plays an important role in the urea cycle, was significantly elevated among ASD cases compared to controls (Fig. 3i). The urea cycle, also known as the ornithine cycle, is the biochemical reaction through which the body excretes excess nitrogen by converting highly toxic ammonia to urea, a pathway that partially takes place in the mitochondria. Deficits in the urea cycle enzyme l-ornithine transcarbamoylase (OTC), which catalyzes the transfer of the carbamoyl group of carbamyl phosphate to ornithine, were previously attributed to autism-like symptoms in a case study of a 4-year-old girl with undiagnosed urea cycle disorder^54^. Interestingly, autism-like symptoms and hyperactivity were no longer apparent after 1 year of treatment. Certainly, it is possible that altered metabolic pathways in ASD may be contributing to the symptoms, severity, or perhaps etiology of the disorder. Collectively, our metabolomics investigation imply reduced glutathione production, dysregulated TCA cycle, mitochondrial dysfunction, and altered nitrogen/amino acid metabolism are associated with ASD.

The developmental conditions under investigation in this study have complex multifactorial etiologies influenced by a number of factors, including gene–environment interactions^3^. Although there is overlap in metabolite levels between the groups, our findings also highlight substantial biochemical differences associated with conditions and elucidate underlying metabolic impairments contributing to clinical phenotypes. To the best of our knowledge, this is the first study to collectively investigate the plasma metabolome of children with ASD, DS, and i-DD cases. Two previous studies have investigated the metabolome of DS cases. One study investigated DS pregnancies (i.e. mothers of children with DS)^55^, and found metabolic alterations during the first trimester of pregnancy associated with DS. Additionally, a more recent study investigated the plasma and urine metabolome in DS cases^56^ and identified impairments of the TCA cycle consistent with our results. Only a few other studies have looked at the blood metabolome in autism^13,57–59^, although numerous others have looked at urine^60–65^. However, many of these other metabolomic studies have reported gastrointestinal (GI) issues as possible confounders in ASD^12,66,67^. Indeed, GI issues are one of the most common medical conditions associated with autism^68^, and are also a common comorbidity in DS^69^. A strength of our study is that we excluded individuals with GI issues and examined a larger sample size compared to similar metabolomics analyses. Had we included children with common GI issues, it could have skewed our metabolite analysis and introduced confounding from GI issues.

This study is unique in that we were able to utilize the existing case–control epidemiologic CHARGE^16^ study to investigate the plasma metabolome and leverage the extensive infrastructure of meta-data available. CHARGE has contributed substantially to the current knowledge of the environmental components relating to autism. For example, it has reported data on associations of maternal metabolic conditons^70^ and prenatal pesticides^71^ with autism. Bridging metabolomics with existing epidemiological studies can help decipher underlying biological pathways, it can speed up analysis as samples are already collected, and facilitate insights through interdisciplinary collaboration. However, utilizing existing data sets for metabolomics analysis also has its caveats. Notably, children in this study did not fast before blood samples were collected, and we did not account for differences in the child’s current diet, which may affect some metabolites. However, we found no association between child’s diagnosis (the predictor variable) and either time fasted (i.e., time since last food intake) or missingness for this variable. Associations were evaluated in our DAG model and a minimum but sufficient set of covariables need to remove confounding was included in our final model. Since child’s current diet is affected by their diagnosis^72,73^ and for reasons of temporality, it would not have played a causal role in its etiology, but instead would be an intermediate on the pathway from diagnosis to metabolite profile. Hence adjustment for child’s current diet would not be appropriate, and could introduce bias into the analyses. Additionally, another limitation in our study is that plasma metabolites were measured after neurodevelopmental diagnosis was made. Therefore, it is not possible to draw conclusions about whether metabolic issues contribute to the onset of any of these conditions. We also did not address maternal metabolomic contributions to in utero or postnatal brain development, although it is plausible that placental transfer or breastfeeding might transmit metabolic influences on early neurodevelopment, potentially explaining observed associations of maternal diabetes with autism or cognitive impairment^70,74–76^. Furthermore, we did not investigate the role of MTHFR genotype and folate status, which may explain alterations in metabolites related to methylation. Lastly, we did not investigate the dual diagnosis of these neurodevelopmental disorders, such as the co-occurrence of ASD and DS which occurs in about 8–38% of DS cases^77–79^.

In conclusion, we identified several metabolic pathways which were affected in children with ASD, DS, and i-DD. Children with DS stood out as having more profound alterations to methylation metabolism, carnitine/*O*-acetylcarnitine, dimethyl sulfone, and myo-inositol. Those with DS or i-DD had similar alterations to the TCA cycle and mitochondrial dysfunction. We also found evidence of altered peripheral mitochondrial dysfunction, glutathione biosynthesis, one-carbon metabolism, urea cycle, and amino acid/nitrogen metabolism in ASD cases. Additional studies investigating developmental delays as metabolic disorders in other study designs could build on the present findings, confirm results, and point to development of potential treatments to mitigate challenging symptoms.

## Supplementary information

Supplementary Figure 1

## Data Availability

The datasets generated and/or analyzed during the current study are available from the corresponding author on reasonable request.

## Code availability

Computer codes used throughout the data analysis are available from the corresponding author on reasonable request.
