# Supplementary material for: Metabolomics analysis of children with autism, idiopathic-developmental delays, and Down syndrome
Source: Transl Psychiatry. 2019 Oct 3;9:243. doi: 10.1038/s41398-019-0578-3 (PMC6776514; doi:10.1038/s41398-019-0578-3)

Supplementary Fig1. Directed acyclic graph (DAG) used in study. Each arrow represents a possible cause-and-effect relationship. Covariates included in final model are in yellow. The association we studied (gray boxes) between neurodevelopmental diagnosis and blood plasma metabolite levels is represented with a question mark.

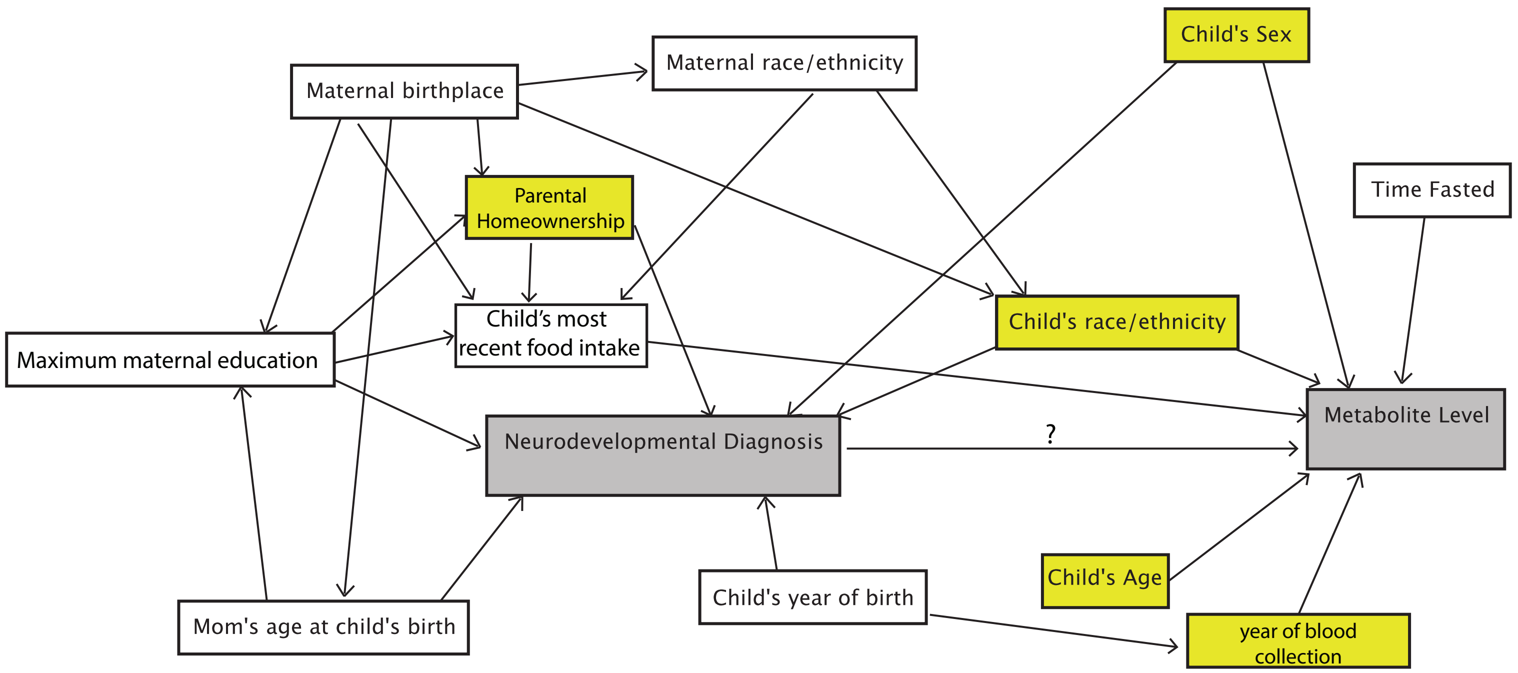

Supplement: Supplementary file 1 — Supplementary Figure 1 [file 41398_2019_578_MOESM1_ESM.pdf]
